# Correlation between corneal thickness, keratometry, age, and differential pressure difference in healthy eyes

**DOI:** 10.1038/s41598-021-83683-2

**Published:** 2021-02-18

**Authors:** Ahmet Colakoglu, Iffet Emel Colakoglu, Cemile Banu Cosar

**Affiliations:** 1 Department of Ophthalmology, Acibadem University School of Medicine, Icerenkoy, 34752 Istanbul, Turkey; 2 Ophthalmology Clinic, Acibadem Bakirkoy Hospital, Istanbul, Turkey

**Keywords:** Biophysics, Medical research

## Abstract

To determine the use of differential pressure difference (DPD), in air-puff differential tonometry, as a potential biomechanical measure of the cornea and elucidate its relationship with the intraocular pressure (IOP), central corneal thickness, corneal curvature, and age. This study comprised 396 eyes from 198 patients and was conducted at Acibadem University, School of Medicine, Department of Ophthalmology, Istanbul, Turkey. The central corneal curvature and refraction of the eyes were measured using an Auto Kerato-Refractometer (KR-1; Topcon Corporation, Tokyo, Japan). IOP and central corneal thickness were measured using a tono-pachymeter (CT-1P; Topcon Corporation, Tokyo, Japan), wherein two separate readings of IOP were obtained using two different modes: 1–30 and 1–60. The difference between these two readings was recorded as the DPD. The factors affecting the DPD were determined by stepwise multiple linear regression analysis. DPD varied over a dynamic range of − 3.0 to + 5.0 mmHg and was weakly correlated with the central corneal thickness (r = 0.115, p < 0.05). DPD showed no significant correlation with IOP 1–30 (p > 0.05). A weak but statistically significant (p < 0.05) positive correlation of DPD was observed with age (r = 0.123), Kavg (r = 0.102), and the CCT (r = 0.115). There was a significant correlation between DPD and Kavg, CCT, and age. There was no significant correlation between DPD and IOP 1–30. Age-related changes in the corneal ultrastructure may be a plausible explanation for the weak positive association between age and DPD. The proposed method may prove a valid non-invasive tool for the evaluation of corneal biomechanics and introduce DPD in the decision-making of routine clinical practice.

## Introduction

Corneal biomechanics evolved as an area for research and development in contemporary ophthalmology and is regarded as a significant contradictory factor for IOP measurement. Comprehending the corneal biomechanical behavior is pertinent to the detection of subclinical keratectasia as well as for the follow-up of ectasia progression.

In vivo evaluation of corneal biomechanics became possible with the advent of the ORA (Ocular Response Analyzer) in 2005. The ORA is an air-pulse tonometer. The Corvis ST (Oculus, Wetzlar, Germany) is also an air-puff tonometer, also approved as a device for assessment of corneal biomechanics. There are new methods, such as the Brillouin optical microscopy, which investigates corneal biomechanics. However, most of the available clinical knowledge is related to the biomechanical response to air-puff tonometry. In addition, in vivo characterization of corneal biomechanics is still influenced by the IOP level in spite of significant progress in this field^1^.

On the other hand, the accuracy of the IOP values is negatively influenced by variation in the ocular rigidity (OR)^2^ and the Goldmann Tonometry is nonexempt from such elastic effects^3^.

To enlighten corneal behavior following physical changes, in vivo evaluation of biomechanical factors is essential. Although several corneal parameters have been previously provided, mainly by the ORA device, it is difficult to draw far-reaching conclusions^4^.

During the popular use of Schiötz tonometry, OR was revealed to be a shared origin of mistake and the differential tonometry was a way of estimating the size of the mistake^5^, but the invasive and time-consuming nature of this tonometry method limited its use in estimating OR in clinical practice.

Young’s modulus (E) is the most pertinent corneal biomechanical feature in tonometric errors, but it impossible to measure these corneal biomechanical features in vivo^6^. Corneal biomechanics have been assessed by assessing the stress–strain and Young’s modulus in isolated corneas^7^. The biophysical parameters contributing to corneal rigidity and elasticity, and maintaining the corneal contour in vivo, are not well known^8^. Current methods for assessing the coefficient of rigidity are unsatisfactory due to variability.

Ophthalmology is in search of methods that will provide information on corneal biomechanics in vivo. The ultrastructure of air-puff tonometry seems to be suitable for this purpose. NCTs, provide a method for IOP measurement, similar to the traditional method of Schiotz tonometry^9^. The non-invasiveness and absent ocular massage effect are the main advantages of NCTs^10^. NCTs are also relatively precise in the assessment of IOP^11^. The IOP measuring range can be switched in 2 steps between "1–30" and "1–60"^10^ so that the eye to be examined with high intraocular pressure (> 30 mmHg) can be measured.

We hypothesized that a set of IOP measurements, both in the 1–30 (low-pressure air pulse) and 1–60 (high-pressure air pulse) mode, emulating the two separate IOP measurements of indentation tonometry with the lower weight (e.g., 5.5 g) and the higher weight (e.g., 10 g), respectively^12^ were required to calculate the average values to produce a differential tonometry method with a NCT. In contrast to the original differential tonometry method, we did not estimate the coefficient of rigidity but used the differential IOP difference as a potential predictor for OR.

This study measured the Differential Pressure Difference (DPD) and investigated potential correlations between DPD and age, sex, and ocular parameters (IOP, CCT, and curvature). The relation of age with DPD paralleled that with the coefficient of ocular rigidity.

## Materials and methods

This study was approved by the institutional review board of Acibadem University and the procedures conformed to the tenets of the Declaration of Helsinki for research involving human subjects. Informed consent was obtained from all subjects. All subjects were recruited from the outpatient section of Acıbadem Atakent University Hospital Eye Clinic. The subjects comprised of 198 healthy volunteers (82 men and 116 women) aged 17 to 74 years who underwent a routine outpatient ophthalmologic examination. Both eyes of 198 healthy subjects were enrolled in the study (396 eyes). This study was conducted between September 2017 and May 2018.

All subjects had no history of corneal diseases. Exclusion criteria were as follows: subjects who are not cooperative in the NCT, those with vision loss who were unable to fixate for this method, history of refractive or intraocular surgery, glaucoma, and use of anti-glaucomatous medication. To minimize the effect of astigmatism on the accurate determination of IOP, subjects with astigmatism of ≥ 3 diopters (as detected by autorefractor keratometer) were excluded.

Before employing the measurements with the tonopachymeter Topcon CT-1P, the visual acuity for each patient was assessed, and the biomicroscopy and non-dilated fundoscopy were employed. First, the corneal curvature and refraction of the eyes were measured with an auto kerato-refractometer (Topcon KR-1; Topcon Corporation, Tokyo, Japan). All the subjects had a 10 min rest before any measurement. The IOP readings were taken with the subjects in a relaxed seated position with both eyes open. Subjects were asked to breathe normally and to quietly fixate on an object behind the technician without blinking. All devices were calibrated at the start of the study. We confirmed that our NCT applied an air-puff to each cornea with the same pressure order. We explained the procedure to the patients in detail.

The CT-1P tonopachymetry was used to measure the corneal thickness through the principle of specular microscopy. The inclinedly emitted light from a narrow slit in the cornea is reflected by the front and backside of the cornea. The reflected light was brought in by the line sensor. The corneal thickness was measured according to the interval between the front and backside reflection images on the line sensor^13^.

To assess IOP using the Topcon CT-1P, four readings were obtained but only the last three readings were averaged to obtain the IOP value for each eye. This method was assimilated to fit the guidelines for the IOP assessment used by the Topcon CT80 air-puff tonometer. After the first air-puff, subsequent puffs are automatically adjusted to the IOP of the subject to minimize the exposure to undue air pressure^10^. The air-puff instant IOP level reflects the variabilities originating from the cardiac and respiratory cycles^14^. Another measurement was performed if any reading was labeled as a low confidence interval or was "out of expected range" (if the difference between the successive readings was over 3 mmHg)^15^.

IOP was assessed using both the 1–30 mode and 1–60 mode tonometry (analogous with low and high Schiötz plunger weights, respectively), and the difference in mode readings was calculated as DPD. By applying more force in a unit time period in 1–60 mode, we aimed to emulate the biophysical conditions created by using 10 gm plunger weight on the corneal surface. That is the reason why the IOP reading with the 1–60 mode always followed the 1–30 mode (emulating 5.5 gm plunger weight).

In order to limit the contributions of the diurnal variations, posture, exercise, ocular movement, straining, and the ingestion of some food materials and/or medicines for the alteration in IOP, the measurements were accomplished in 15 min^16^. The mean of three CCT readings within an SD of ± 5 µm was calculated for both eyes. All readings were taken by the same technician.

### Sample size

The sample size in this clinical trial was estimated using the PASS 13 software (NCSS, LLC, Kaysville, Utah, United States). Assuming a weak correlation (coefficient, r =  + 0.20 or r = − 0.20) between the DPD and the other variables (i.e., age, CCT, and average keratometric value), we calculated the actual total sample size required to achieve 80% power and two-sided significance level ɑ of 0.05 to be 193.

### Statistical analyses

IBM SPSS Statistics version 25.0 software (IBM Corporation, Armonk, New York, United States) was used to analyze the variables. Kolmogorov–Smirnov with Lilliefors correction and Shapiro–Wilk normality tests were used to confirm the normal distribution of the data and Levene's test was used to check for the homogeneity of variances. The non-parametric Mann–Whitney U test with the Monte Carlo simulation technique was used to compare the two independent groups in terms of quantitative data, whereas the independent samples bootstrapped t-test was used as the parametric method.

The partial correlation test was performed to calculate the correlation between the two variables while controlling for the effects of sex. To reveal the causality between the dependent and independent variables in the form of representing the mathematical model, linear regression analysis as one of the machine learning approaches was tested with a forward stepwise method.

Quantitative variables were expressed as mean ± SD (standard deviation), median range (maximum-minimum), and categorical variables as n (%) in the tables. Variables were examined at a 95% confidence level and a p-value of less than 0.05 was considered to be statistically significant.

For statistical or mathematical modeling, applied to reveal the causal relationships between the dependent and independent variables, one of the machine learning methods, a standard model was used. Linear regression analysis, as one of the machine learning approaches, was tested with a forward stepwise method. Automatic Data Preparation steps together with adjustment of measurement level, outlier and missing value handling, supervised merging, and boosting / bagging were applied to increase the predictive power. The model selection criterion used in Forward stepwise selection method was AICc, an information criterion.

### Ethics approval and consent to participate

This research involves human participants and human data. The informed consent obtained was verbal. Obtaining verbal consent was seen as way of reducing the stress and demands placed on our outpatient clinic visitors who, as a result of the air puff procedure of non-contact tonometry, had been experiencing anxiety having the potential leading to an overestimation of IOP readings. This view was reinforced by the head of the Department Ophthalmology who concluded that, "in this situation at least, we should much rather get verbal consent as far as the non-invasive, patient-friendly nature of non-contact tonometry is concerned”.It has been performed in accordance with the Declaration of Helsinki and has been approved by the institutional ethics committee of Acibadem University School of Medicine.

### Consent to publish

There are no details on individuals reported within the manuscript.

## Results

The demographic and ocular features of the study population are presented in Tables 1 and 2 respectively.

**Table 1:** Demographic features of the study population (n = 198).

Gender	n	%
Female	116	58,60%
Male	82	41,40%

	Mean ± SD	Median (min/max)
Age (years)	43.83 ± 14.32	43 (17 / 74)

*SD* standard deviation, *min* minimum, *max*:maximum.

**Table 2:** Ocular features of the study population and eye laterality results.

	Right (n = 198)	Left (n = 198)	P
	Mean ± SD	Mean ± SD	
CCT	547.73 ± 32.09	549.07 ± 32.32	0.005 p^†^
IOP 1–30	16.03 ± 2.71	16.23 ± 2.71	0.034 p^†^
IOP 1–60	18.15 ± 3.01	18.26 ± 3.05	0.230 p
K_avg_	43.51 ± 1.48	43.55 ± 1.51	
DPD	2.12 ± 1.19	2.04 ± 1.15	

	Median (min/max)	Median (min/max)	
CCT	547 (468/622)	546 (462/632)	
IOP 1–30	15.7 (8.70/23)	16 (7.30/23.30)	
IOP 1–60	18 (10/26)	18 (9/27)	
K_avg_	43.38 (39.38/47.38)	43.63 (39.25/47.63)	0.093 w
DPD	2.20 (− 3/5)	2 (− 3/4.70)	0.263 w

*p* paired samples T test (bootstrap), *w* Wilcoxon sign ranks test (Monte Carlo), *SD *standard deviation, *min* minimum, *max* maximum, *IOP 1–30* intraocular pressure in mm Hg (1–30 mode), *IOP 1–60* intraocular pressure in mm Hg (1–60 mode), *DPD* differential pressure difference in mm Hg, *K*_*avg*_ average keratometric value in diopters, *CCT* central corneal thickness in micrometers.

There were no statistically significant differences in any of the parameters between men and women (Table 3).

**Table 3:** Differences in ocular parameters between sexes.

	Right		P	Left		P
	Female (n = 116)	Male (n = 82)		Female (n = 116)	Male (n = 82)	
Age mean ± SD. (min/max)	44.90 ± 15.08 (17/74)	42.33 ± 13.13 (18/74)	0.216 t	44.90 ± 15.08 (17/74)	42.33 ± 13.13 (18/74)	0.216 t
DPD median (min/max)	2.3 (− 3/5)	2 (− 0.5/5)	0.428 u	2 (− 3/4.4)	2 (− 1/4.7)	0.277 u
IOP 1–30 median (min/max)	15.7 (8.7/23)	15.5 (10.3/22.7)	0.967 u	16 (7.3/23)	15.85 (10.3/23.3)	0.461 u
IOP 1–60 mean ± SD	18.25 ± 3.08	18.00 ± 2.92	0.989 t	18.26 ± 2.93	18.26 ± 3.23	0.585 t
K_avg_ mean (min/max)	43.75 (40.12/46.87)	43.37 (39.25/47.62)	0.117 u	43.62 (40.12 /47.37)	43.25 (39.37/47)	0.055 u
CCT median (min/max)	549 (468/616)	544.5 (476/622)	0.804 u	548.5 (462/632)	545.5 (482/625)	0.786 u

*t* ındependent samples T test (bootstrap), *u* Mann Whitney U test (Monte Carlo), *SD* standard deviation, *min* minimum, *max *maximum, *IOP 1–30* intraocular pressure in mm Hg (1–30 mode), *IOP 1–60* intraocular pressure in mm Hg (1–60 mode), *DPD* differential pressure difference in mm Hg, *K*_*avg*_ average keratometric value in diopters, *CCT* central corneal thickness in micrometers.

Table 2 shows the median of the DPD and the Kavg. Mean CCT and IOP 1–30 values, which were both higher in the left eyes than the right eyes. These results were statistically significant but not considered to be clinically relevant. DPD showed no significant differences with laterality (Table 2).

The DPD increased significantly with the increasing age, IOP 1–60, Kavg, and CCT (Table 4). There was a moderate positive correlation between the DPD and IOP 1–60 (r = 0.448, p   < 0.001). Meanwhile, there was a weak positive correlation between the DPD and the CCT (r = 0.115, p = 0.022), Kavg (r = 0.102, p = 0.043), and age (r = 0.123, p = 0.014). No correlation was observed between the DPD and IOP 1–30 (r = 0.069, p = 0.170) (Table 4).

**Table 4:** Correlations between ocular parameters.

		Total eye (n = 396)		Right (n = 198)		Left (n = 198)	
		r	P	r	P	r	P
**DPD**							
	IOP 1–30	0.069	0.17	0.04	0.572	0.102	0.155
Mod	IOP 1–60	0.448	< 0.001^†^	0.432	< 0.001^†^	0.466	< 0.001^†^
Low	K avg	0.102	0.043^†^	0.049	0.497	0.157	0.027
Low	CCT	0.115	0.022^†^	0.055	0.44	0.178	0.012^†^
Low	Age	0.123	0.014^†^	0.14	0.049^†^	0.105	0.14
**K**_**avg**_							
Age		0.102	0.043^†^	0.049	0.497	0.157	0.027^†^
IOP 1–30		− 0.093	0.065	− 0.09	0.209	− 0.098	0.172
IOP 1–60		− 0.044	0.382	− 0.062	0.388	− 0.028	0.697
CCT		− 0.148	0.003^†^	− 0.137	0.056	− 0.16	0.024^†^
Age		0.089	0.078	0.094	0.189	0.083	0.244
**CCT**							
DPD		0.115	0.022^†^	0.055	0.44	0.178	0.012^†^
IOP 1–30		0.497	< 0.001^†^	0.518	< 0.001^†^	0.476	< 0.001^†^
IOP 1–60		0.49	< 0.001^†^	0.49	< 0.001^†^	0.49	< 0.001^†^
K_avg_		− 0.148	0.003^†^	− 0.137	0.056	− 0.16	0.024^†^
Age		0.075	0.137	0.068	0.342	0.082	0.253

Partial correlation test, controlled for the effects of sex.

*r* correlation coefficient,* IOP 1–30* intraocular pressure in mm Hg (1–30 mode), *IOP 1–60* intraocular pressure in mm Hg (1–60 mode), *DPD* differential pressure difference in mm Hg, *K*_*avg*_ average keratometric value in diopters, *CCT* central corneal thickness in micrometers.

Low negative correlation coefficient: − 0.29 to − 0.10.

Moderate negative correlation coefficient: − 0.49 to − 0.30.

High negative correlation coefficient: − 0.50 to – 1.00.

Low positive correlation coefficient: 0.10 to 0.29.

Moderate positive correlation coefficient: 0.30 to 0.49.

High positive correlation coefficient: 0.50 to 1.00.

Data from all eyes were entered into a machine learning model with linear regression; forward stepwise as the wrapper method. The model included the age in years (categorized according to the most informative cutoffs of ≤ 40), Kavg, and CCT as the independent variables. The dependent variable was DPD (Table 5).

**Table 5:** Machine learning model with linear regression results.

Dependent variable: DPD		B (SE)	P value	Significance	R^2^	P (model)
**Total eye**						
K_avg_		0.092 (0.039)	0.02	0.344	0.032	0.004
CCT		0.004 (0.002)	0.023	0.327		
Age (≤ 40)		− 0.231 (0.117)	0.049	0.244		
Constant		− 4.479 (2.115)	0.035			

Machine learning: linear regression (forward stepwise).

*B* regression coefficients, *SE* standard error, *R*^*2*^ explanation level of the postulated model (coefficient of determination).

Explanation of the postulated model is likely not important and therefore omitted (R^2^ = 0.032) even though it was statistically significant at a certain level (p = 0.004).

## Discussion

This study determined the use of DPD in air-puff differential tonometry, as a potential biomechanical measure of the cornea and elucidated its relationship with IOP, central corneal thickness, corneal curvature, and age. We propose a method that may provide a valid non-invasive tool for the evaluation of corneal biomechanics and introduce the use of DPD for decision-making in routine clinical practice.

Low pulse IOP reading (1–30 mode) was always the first to be done before the high pulse one (1–60 mode). This approach was necessary to prevent predilections due to possible decrements in IOP levels which might have been caused by the IOP-lowering influence aqueous massage on repetitive high-pressure air pulse readings in the 1–60 mode^17^. Since the magnitude and the rate at which the air-puff is applied is higher and faster in the 1–60 mode, the production of a greater massaging effect is expected. Potential results of eye massaging have been observed with the GAT but have been irrelevant with the low-pressure air pulse, i.e., 1–30 mode non-contact tonometry^11^. Nevertheless, it is still possible that successive IOP readings may have led to reduced measurement values due to the egress of aqueous humor from the eye during the air pulses and due to the tissue preconditioning^18^.

Until recently, reports on corneal biomechanical features has been exclusively on the eye bank corneas^19,20^. In vivo corneal biomechanics studies are required to elucidate corneal tissue behavior during physical changes. Despite many studies, mainly using the ORA instrument, its properties remain unclear^4^. Noninvasive evaluation of corneal biomechanics does not yet provide elastic modulus^21^. Detorakis et al. proposed a tool to measure OR based on Friedenwald’s principle^3^. This method required two different tonometers, the GAT and Dynamic Contour Tonometry, for IOP readings. Although in the present study, we could not measure the coefficient of rigidity, in contrast to the above-mentioned study, we employed a noncontact air-puff technique in just one device, thereby reducing cost.

We are not able to determine whether DPD reflected the degree of corneal viscosity, corneal elasticity, or corneal rigidity. DPD may be an in vivo reflector of the corneal rigidity as far as the differential tonometry method employed for its assessment is concerned.

It was well known that the corneal biomechanics affect ORA IOP measurements. The corneal constant factor (CCF) has been proposed to reduce this effect^22^, which was in a positive and negative correlation with CCT and age, respectively^4^. The correlation between CH and CCT or corneal radius is very weak^23^. Furthermore, the total corneal rigidity is lower than normal in the case of low corneal resistance factor (CRF)^24^. Interindividual comparison of hysteresis values is not feasible as they represent the effect of a set of biomechanical factors^25^. Neither of CH and CRF can be regarded as corneal features because they are reactions that are peculiar to the ORA assessment procedure^26^. Unfortunately, the validity and definitions of CH, CRF, and ORAcc have not been cogently established^26^ and further studies are required to investigate the exact representation by these three parameters (CH, CCF, and CRF)^27^.

According to Simonini et al. the interpretation of the data obtained by the ORA may not yet play a key role in clinical practice^28^. The ORA CH may not be a true representation of hysteresis of tissues^25^. The Corvis ST is more advantageous than the ORA as it provides a direct deformation quantification^29^.

Nevertheless, the entire assumption behind the Corvis ST has been unpublished until now^30^. The uniqueness of the Corvis ST is its ability to provide a quantitative measure of stiffness without any advance assumptions^31^. The Corvis ST is not immune to corneal biomechanical effects^30^.

Thin cornea and low IOP are associated with the less stiff cornea^32^. Ocular biomechanics are characterized by the Corvis ST-produced factors, which are affected by corneal thickness, and curvatures of the anterior and posterior surfaces^33^. The relation between biomechanical factors produced by Corvis ST and the conventional biomechanical features is unclear^32^. The above-mentioned air-puff measured parameters do not directly reflect the actual biomechanical parameters but only the geometrical ones^34^, and both the Corvis ST and ORA instruments are unable to provide a direct estimation of corneal elasticity^35^. In addition, we cannot compare the results among the ORA and Corvis ST^29^.

Tejwani et al. found CH and CRF exhibit significant differences between the left and right eyes but not the DA of the Corvis ST, contradicting the general acceptance that the eyes of an individual are biomechanically similar^36^. In the present study, the median value of DPD was 2.20 mmHg for the right eyes and 2.00 mmHg for the left eyes. DPD was found not statistically significantly different between the right and left eye (p = 0.263).

Hysteresis and the CRF and CCT may be associated^37^. A significant correlation between the pachymetric results and the relevant Corvis ST parameters has been observed^33^.

Pallikaris et al. did not report any significant correlation between the coefficient of OR and CCT^38^. In our study, we detected a weak positive correlation between the DPD and CCT (r = 0.115, p = 0.022). This result was similar to the previously reported relationship between the CCT and corneal rigidity.

If the stiffness increases, the coefficient of OR also increases, especially in aging people^39^. An age-related increase in corneal stiffness has been demonstrated in a recent study employing a button inflation investigation in the ex vivo corneal tissue^39^. Corneal biomechanics change with aging and elastic modulus approximately doubles between the ages of 25 and 100^40^.

Waveform analyses of air-puff deformation with Corvis ST presented corneal stiffness and extraocular tissue stiffness (EOS). A significant positive correlation has been reported between EOS and age^29^. However, Lau et al. claimed that age did not significantly affect any of the Corvis ST factors^6^. In contrast to the null or negative correlation between hysteresis and aging, the present study demonstrated a statistically significant positive correlation between the DPD and age, albeit a weak one (r = 0.123, p = 0.014). Nevertheless, this relationship parallels the established relationship between OR and age. The effect of cross-linking therapy on DPD should be investigated in a large sample study to note any possible increase in the values of DPD paralleling those in the coefficients of OR. Sedaghat et al. found no significant change in the biomechanical corneal properties (i.e., CH and CRF) following cross-linking procedure^41^.

Several methodologies that assess OR have several disadvantages such as being too invasive, have low precision, reproducibility or technical intricacies^42^. It is important to note that OR is a macroscopic parameter addressing the pseudo-static pressure–volume alterations. Therefore, relatively fast alterations, i.e., those occurring during corneal deformation by an air jet or the cardiac cycle, may reflect a different pressure–volume relationship. In this case, consideration of the viscoelastic properties of the ocular coating is essential^43^. Nevertheless, we speculate that by using NCT in our study, we were able to mimic the "differential tonometry" method of paired Schiötz tonometry proposed by Friedenwald.

The cornea of male subjects is reportedly more rigid than those of female subjects^44^, Contrasting with the results of earlier studies on OR and sex,we did not observe a statistically significant difference between the female and male DPDs in the present study,The absence of statistical significance may merely be a manifestation of inadequate statistical power to detect a relevant association as the statistical power of our study was based on the DPD.

A greater deformation was required to applanate a steeper cornea, resulting in a higher ORA measured IOP level; however, the association between the corneal steepness and the parameters measured by the ORA parameters was statistically insignificant^45^.

In some studies, corneal astigmatism was found to be negatively correlated with CH and CRF. Some studies do not support the association between the keratometry and Corvis ST parameters^33^. Nemeth et al. concluded that the corneal curvature values may affect the measured parameters of the Corvis ST device^46^.

In the present study, Kavg had a weak positive correlation with the DPD (r = 0.102; p = 0.043) while the DPD exhibited the same association between Kavg and the corneal stiffness.

NCT is more affected by CCT than the GAT, which may be justified by the corneal viscoelastic property, where the rate of impingement of force affects the stiffness^47^.

As expected, our study, depending on an air-puff method, identified that CCT was positively and moderately correlated with both IOP1-30 (r = 0.497, p < 0.001) and IOP1-60 (r = 0.490, p < 0.001). This result paralleled the findings of a previous study^48^. The same relative biomechanical relationship applies to both the ORA and Corvis ST devices: the stiffer eye will deform and move slower, and recover quicker^49^.

Although both the ORA and Corvis ST devices study deformation of the corneal layer in response to an intense air pulse, the parameters acquired by these two instruments are not comparable^33,50^. We believe that the DPD, although obtained by a method based on the air-puff technique, is a biophysical parameter that cannot be compared to the ORA and Corvis ST parameters. The DPD parameter could be compared with the coefficient of rigidity as far as the differential tonometry employed for both concepts is concerned.

In the present study, a positive correlation was observed between the DPD and IOP 1–30, which was statistically insignificant (r = 0.069, p = 0.170). Corneal Young’s modulus also exhibits variation with IOP^27^. Studies examining the CH and CRF have suggested an association with IOP; however, some report no association with these parameters^18^. In another study, CH and IOP were weakly correlated^20^. A recent study revealed a significant but weak negative correlation between the IOP and CH^23^.

An intrinsically stiff cornea exposed to low IOP levels may exhibit a softer response than an intrinsically soft cornea exposed to high IOP^9^. In vivo values of corneal Young’s modulus changes with real IOP, in a way that higher real IOP levels may force the cornea to behave like a stiffer tissue^51^.

The incremental rate of air pressure and maximum magnitude reached by the air-puff force will affect measured IOP levels. A stiffer corneal response will be produced by a faster strain rate^49^. The implementation rate of force has to increase with intraocular pressure and the hysteresis may be affected^22^. A viscoelastic material would be expected to be more resistant to the deformation if the force was implemented in a shorter period. This may explain the more rigid behavior of the cornea using a NCT than the GAT^26^. Another explanation could be that NCT acts on a larger corneal surface and is more susceptible to variations in CCT^26^.

The present study demonstrated different results for the same IOP levels when assessed in two different measuring modes The average IOP1-60 (18.20 + /− 3.02 mmHg) was higher than the IOP1-30 (16.13 + /− 2.71 mmHg). Regarding this difference, we propose that the more intense force of air used for 1–60 mode most probably lead to a stiffer response from the cornea and resulted in higher levels of IOP than those measured in the 1–30 mode.

Among the multiple techniques which have emerged recently, the most common approach for measuring corneal biomechanics is to employ an air puff perturbation and analyzing corneal deformation response using topographers, tonometers, and optical coherence tomographers (for example, ORA, Corvis ST)^52^. The CorVis ST device may be insensitive to detect the age-related change in corneal biomechanics^53^. Our DPD method also employs air puff perturbation but no other complex instrumentation or computation. DPD can be readily incorporated into the parameter sets generated by ORA and CorvisST. This facility may ease the study of DPD as a new potential parameter. Another advantage of DPD compared with the biomechanical parameters provided by the commonly used machines such as ORA is that DPD displays positive correlation with age paralleling the correlation of ocular rigidity with age.

Our study has a small sample size, and subjects were not inquired of previous systemic diseases such as Graves' disease, that may have affected the corneal biomechanics^54^. Another limitation is that we did not compare the CCT values from the tono-pachymeter with those from the ultrasound pachymeter.

## Conclusions

We presented a parameter, DPD, utilized to evaluate the in vivo biomechanical response of normal cornea derived from tono-pachymeter-derived measurements. DPD can be used as a potential parameter following modification of tono-pachymeter software and simply converting the device into a tonopacho-biomechanometer. The usefulness of DPD in determining corneal pathologies is yet to be assessed. However, the method employed to obtain DPD by air-puff tonometry is user-friendly, low cost, and of non-invasive nature. DPD may be an alternative and potentially useful biophysical variable for evaluating ocular bio-properties. Further studies are required to infer what biomechanical properties are delineated by this parameter and propose more clinical applications based on its use.

## Data Availability

The datasets used and/or analysed during the current study are available from the corresponding author on reasonable request.

## Abbreviations

DPD: Differential pressure difference
IOP: Intraocular pressure
NCT: Non-contact tonometer
OR: Ocular rigidity
ORA: Ocular response analyzer
E: Young’s modulus
CCT: Central corneal thickness
K_avg_: Average central corneal curvature
SD: Standard deviation
GAT: Goldmann applanation tonometry
CH: Corneal hysteresis
CCF: Corneal constant factor
CRF: Corneal resistance factor
cc: Corneal compensated
DA: Deformation amplitude
EOS: Extraocular tissue stiffness
